# Secretion of glucagon, GLP-1 and GIP may be affected by circadian rhythm in healthy males

**DOI:** 10.1186/s12902-024-01566-9

**Published:** 2024-03-14

**Authors:** Dorte B. Zilstorff, Michael M. Richter, Jens Hannibal, Henrik L. Jørgensen, Henriette P. Sennels, Nicolai J. Wewer Albrechtsen

**Affiliations:** 1 Department of Clinical Biochemistry, Copenhagen University Hospital - Bispebjerg Hospital, Copenhagen, Denmark; 2 NNF Center for Protein Research, Faculty of Health and Medical Sciences, University of Copenhagen, Copenhagen, Denmark; 3 Department of Clinical Medicine, Faculty of Health and Medical Sciences, University of Copenhagen, Copenhagen, Denmark; 4 Department of Clinical Biochemistry, Copenhagen University Hospital – Hvidovre, Hvidovre, Denmark

**Keywords:** Circadian rhythm, Glucagon, GLP-1, GIP, Incretin hormones, Glucagon stimulation, Metabolism

## Abstract

**Background:**

Glucagon is secreted from pancreatic alpha cells in response to low blood glucose and increases hepatic glucose production. Furthermore, glucagon enhances hepatic protein and lipid metabolism during a mixed meal. Glucagon-like peptide-1 (GLP-1) and glucose-dependent insulinotropic polypeptide (GIP) are secreted from gut endocrine cells during meals and control glucose homeostasis by potentiating insulin secretion and inhibiting food intake.

Both glucose homeostasis and food intake have been reported to be affected by circadian rhythms and vice versa. In this study, we investigated whether the secretion of glucagon, GLP-1 and GIP was affected by circadian rhythms.

**Methods:**

A total of 24 healthy men with regular sleep schedules were examined for 24 h at the hospital ward with 15 h of wakefulness and 9 h of sleep. Food intake was standardized, and blood samples were obtained every third hour. Plasma concentrations of glucagon, GLP-1 and GIP were measured, and data were analyzed by rhythmometric statistical methods. Available data on plasma glucose and plasma C-peptide were also included.

**Results:**

Plasma concentrations of glucagon, GLP-1, GIP, C-peptide and glucose fluctuated with a diurnal 24-h rhythm, with the highest levels during the day and the lowest levels during the night: glucagon (*p* < 0.0001, peak time 18:26 h), GLP-1 (*p* < 0.0001, peak time 17:28 h), GIP (*p* < 0.0001, peak time 18:01 h), C-peptide (*p* < 0.0001, peak time 17.59 h), and glucose (*p* < 0.0001, peak time 23:26 h). As expected, we found significant correlations between plasma concentrations of C-peptide and GLP-1 and GIP but did not find correlations between glucose concentrations and concentrations of glucagon, GLP-1 and GIP.

**Conclusions:**

Our results demonstrate that under meal conditions that are similar to that of many free-living individuals, plasma concentrations of glucagon, GLP-1 and GIP were observed to be higher during daytime and evening than overnight. These findings underpin disturbed circadian rhythm as a potential risk factor for diabetes and obesity.

**Trial registration:**

ClinicalTrials.gov Identifier: NCT06166368. Registered 12 December 2023.

## Background

Glucagon is secreted from pancreatic alpha cells and increases blood glucose but also influences hepatic protein and lipid metabolism during mixed meals [1]. Especially amino acids stimulate the secretion of glucagon from the alpha cell [2]. Furthermore, glucagon also possesses beneficial effects on multiple organs including the pancreas, kidney, heart, and brain [1]. Increased plasma glucagon (hyperglucagonemia) contributes directly to hyperglycemia in patients with type 2 diabetes (T2D) [3].

The incretin hormones glucagon-like peptide-1 (GLP-1) and glucose-dependent insulinotropic polypeptide (GIP) potentiate glucose-induced insulin secretion during a meal, an effect called the incretin effect [3]. GLP-1 furthermore promotes satiety and suppress appetite [4]. GLP-1 and GIP are secreted from the small intestine during meals. GLP-1 is mainly secreted by intestinal L-cells and GIP by intestinal K-cells located along the small intestine. The secretion of the incretin hormones varies with the distribution of macronutrients in the meal and size of the meal [4]. The incretin effect is severely reduced in patients with T2D, and co-agonists of these hormones are now approved and used as medication for T2D and obesity [5].

Glucagon, GLP-1 and GIP circulate in the blood with concentrations in the low picomolar range. Previously, accurate measurements of glucagon, GLP-1 and GIP have been difficult, but with the development of validated enzyme-linked immunosorbent assays (ELISA) this has become possible [6, 7]. Plasma levels of glucagon, GLP-1, and GIP are used in numerous clinical trials as markers for metabolic health [6, 7].

Circadian rhythm in physiology and behavior is in mammals driven by the endogenous biological master clock located in the hypothalamic suprachiasmatic nuclei and cycling clocks in all peripheral tissues [8, 9]. Circadian rhythm is modulated by daylight, food intake, environmental temperature, and physical activity [10]. Modern living standards such as prolonged artificial lightning, short sleep duration, shift work, sedentary lifestyle, intestinal flora, and high-calorie food intake may therefore influence circadian rhythm [11]. Disruption of circadian rhythms is known to be a risk factor for metabolic disorders, including impaired insulin secretion, abnormal glucose tolerance, obesity and T2D [12, 13].

It is well known that the insulin response to meal ingestion is more rapid in the morning than in the afternoon [14, 15] and as a potential explanation it has been suggested that both GLP-1 and GIP may exhibit a circadian rhythm in both glucose-tolerant and diabetic obese patients [16]. No clear diurnal pattern of the incretin hormones has been found [17–20], but it has been shown that the early release of GLP-1 and GIP are more pronounced in the morning than in the afternoon [21]. To our knowledge, a circadian rhythm of glucagon has not yet been shown.

In this study, we investigated whether the secretion of glucagon, GLP-1 and GIP show a diurnal rhythm in 24 healthy young males, under meal conditions that are similar to that of many free-living individuals, using validated measurement techniques. To assess the associations to changes in plasma levels of glucose and beta cell function we included previously published data on plasma C-peptide, glucose and HbA1c measurements [22].

## Methods

### Approvals

The study was conducted in accordance with the Declaration of Helsinki and approved by the Scientific Ethics committee of the Capital region of Denmark (protocol number H-B-2008-011) registered with the Danish Data Protection Agency (journal number 2008-41-1821). The study is registered at clinicaltrial.gov (NCT06166368). The study protocol and data related to the study primary outcome have previously been published [23].

### Study participants

A total of 24 men were included in the study. The inclusion and exclusion criteria were assessed at an interview by questioning. The inclusion criteria included: male gender, age between 18–45 years-old, medically healthy, non-to normal alcohol intake (0–10 drinks per week) and no use of illicit drugs. The subjects were excluded from the study if they suffered from acute or chronic medical diseases, had worked nightshifts the last 14 days before the study, been transatlantic traveling the last 14 days before the study, had been smoking the last 14 days before the study, had been binge drinking (more than 5 drinks on one occasion) 14 days before the study, had plasma hemoglobin < 8.0 mmol/L (12.9 g/dL) or had a body mass index (BMI) < 18.5 kg/m^2^ or > 24.9 kg/m^2^. Previously published data on HbA1c [22] were included to show that none of the participants suffered from diabetes. When systematically questioned none of the participants had sleep complaints and they all had a normal sleep–wake pattern. The subjects were excluded from the study if they had an irregular sleep–wake pattern, didn’t sleep 7–8 h per night or were extreme morning- or evening types [24]. Melatonin concentrations every third hour from the healthy subjects have previously been shown to validate the circadian rhythm of the participants [23].

The volunteers were recruited by advertisements at University of Copenhagen.

### Study design

**Fig. 1:**
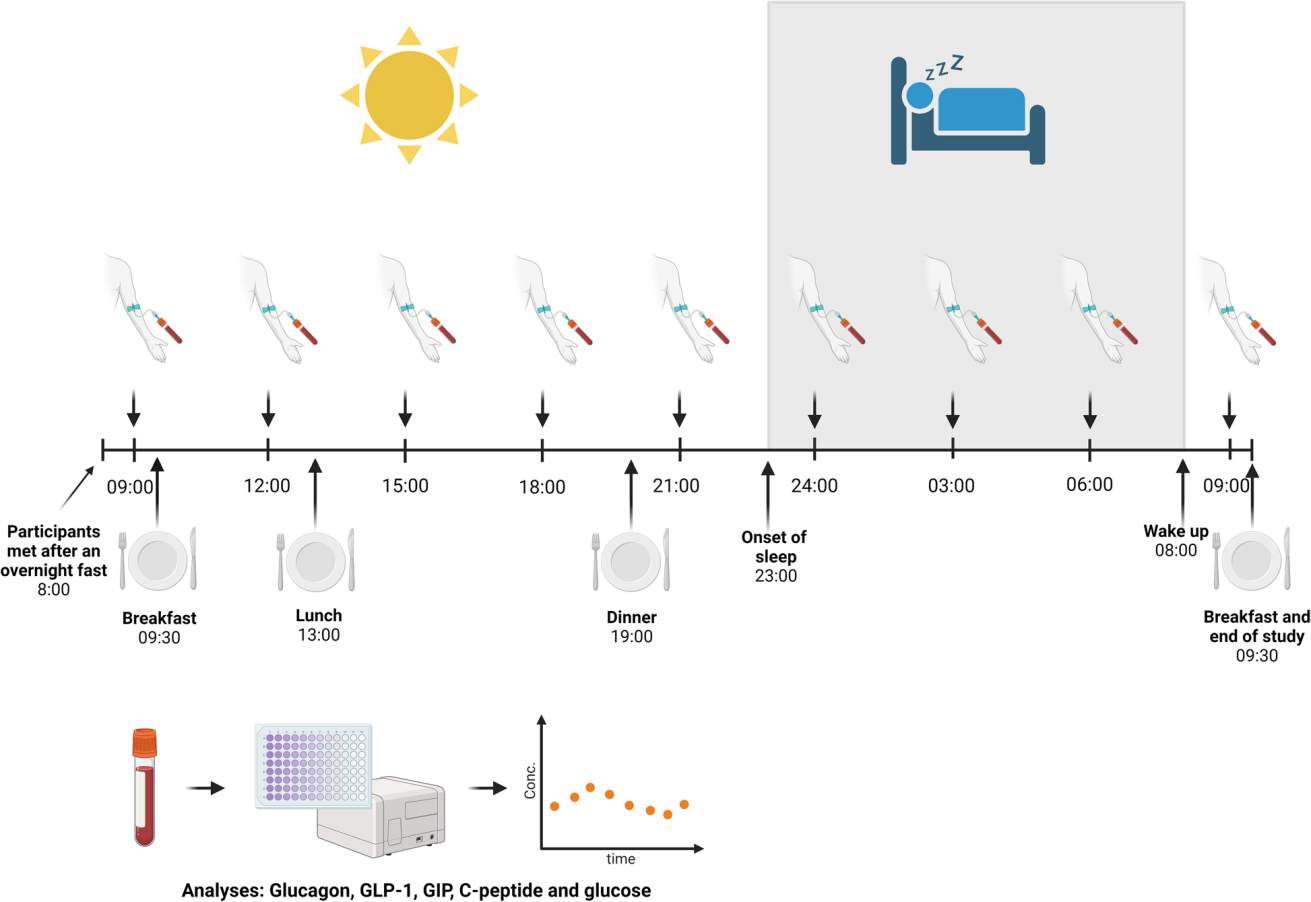
Study design. 24 healthy males met after an overnight fast at 8:00 h at the hospital ward. At 9:00 h and every third hour blood samples were drawn. Breakfast was served at 9:30 h (846 kcal, carbohydrate 47.8 E%, protein 16.3 E%, fat 35.1 E%), lunch was served at 13:00 h (636 kcal, carbohydrate 58.8 E%, protein 19.9 E%, fat 21.1 E%) and dinner was served at 19:00 h (841 kcal, carbohydrate 45.3 E%, protein 19.6 E%, fat 35.1 E%). From 23:00 h to 8:00 h the participants had a 9-h sleep opportunity. The study was carried out on three different days, each time with eight participants. Blood analyses for glucagon, GLP-1, GIP, C-peptide, and glucose were performed subsequently in batch analyses. The illustration was created with www.biorender.com

After an overnight fast, study participants met at the hospital ward at 8:00 h. At 9:00 h the first blood sample was drawn. Blood sampling was repeated every third hour at 12:00 h, 15:00 h, 18:00 h, 21:00 h, 24:00 h, 03:00 h, 06:00 h and 09:00 h the next morning. At 9:30 h, 13:00 h and 19:00 h breakfast, lunch and dinner were served respectively. Throughout the study, the participants refrained from: drinking alcohol, using tobacco, eating candy, and performing strenuous exercise. Activities during the awake time included reading, watching television, low-intensity walking and low-intensity activity while sitting. From 23:00 h to 08:00 h the participants had a 9-h sleep opportunity in the dark (Fig. 1). The subjects were monitored by awake guards during the night who reported that all the participants slept through the night and none of the participants awoke during blood sampling [23].

The study was carried out on three different days, each time with eight participants.

### Meal composition

The meals were standardized in such a way that all the subjects were offered the same meals, however, no measurements of the amount of each offered meal the subjects ate were made. Therefore, an estimation of average calorie intake and distribution of macronutrients per meal for an average male person were calculated using a diet calculator (Vitakost, Denmark).

All meals were mixed meals: breakfast (846 kcal, carbohydrate 47.8% energy percent (E%), protein 16.3 E%, fat 35.1 E%), lunch (636 kcal, carbohydrate 58.8 E%, protein 19.9 E%, fat 21.1 E%) and dinner (841 kcal, carbohydrate 45.3 E%, protein 19.6 E%, fat 35.1 E%).

### Blood sampling

Every third hour each participant had blood samples drawn (9 time-points in total). At the first blood sampling at 09:00 h the participants were fasting from 22:00 h. Two subjects did not fast overnight and therefore two measurements at the timepoint 9:00 h were excluded from analyses. Blood samples were drawn by cubital vein-puncture with minimal use of tourniquet. During the day blood samples were drawn after 10 min of rest in sitting position with horizontal legs. During the sleep period the blood samples were drawn guided by red light (19 lux) and minimal disturbance to the participants [23].

Blood samples for glucagon, GLP-1 and GIP were drawn in K_3_EDTA (ethylene diamine tetraacetic acid) plasma tubes (Greiner Bio-one, Frickenhausen, Germany) and centrifugated immediately. The plasma was stored for -80 °C until analysis. Preanalytical considerations were followed as previously described [4, 7].

Blood samples for C-peptide were drawn in serum clot activator tubes coated with microscopic silica particles (Greiner Bio-one, Frickenhausen, Germany) and were set to clot for 30 min at room temperature before centrifugation. The serum was stored at -80 °C until analysis [22].

Blood samples for glucose were drawn in lithium heparin tubes (Greiner Bio-one, Frickenhausen, Germany) and centrifuged. The samples were analyzed immediately after sampling [22].

Blood samples for HbA1c were drawn in K_3_EDTA plasma tubes (Greiner Bio-one, Frickenhausen, Germany) and stored at -20 °C until analysis [22].

### Biochemical analysis

Plasma glucagon concentrations were measured with colorimetric enzyme-linked immunosorbent assays (ELISA) protocols from Mercodia (Catalog number 10-1271-01, Mercodia AB, Uppsala, Sweden). Plasma concentrations of GLP-1 and GIP were measured with chemiluminescent ELISA protocols from Mercodia (Catalog number 10-1278-01 and 10-1258-01 respectively, Mercodia AB, Uppsala, Sweden). For all three analyses intraassay- and interassay variation was below 15%.

Glucose concentrations were determined on the Vitros 5.1 FS (Ortho-Clinical Diagnostics, Rochester, NY USA) by colorimetric slide technology with an interassay variation below 4.1% and an intraassay variation below 2.1% [22].

C-peptide concentrations were determined on the Cobas e411 (Roche Diagnostics, Basel, Switzerland) by electro-chemiluminescence immunoassays with an interassay variation of 2.3% and an intraassay variation of 0.6% [22].

HbA1c concentrations were determined on the Tosoh HLC-723 G8 (Tosoh Coporation, Tokyo, Japan) by high performance liquid chromatography (HPLC) with an interassay variation of 0.7% and an intraassay variation of 0.3% [22].

### Data analysis

Data were analyzed for a circadian rhythm using the methods for cosinor-rythmometry [25]. The 24-h rhythms were characterized by the rhythm parameters: mesor (rhythm adjusted average about which oscillation occurs), amplitude (half the difference between the highest and lowest value of the fitted cosinor curve) and time of peak. Peak times and nadir times were 12 h apart due to the symmetric cosine-curve model used to analyze the data.

The fasting blood samples at 9:00 h for two subjects were excluded from the analyses as they were not fasted. The measurements at the remaining eight timepoints were included in the analyses. Analyses were also performed with these two subjects excluded completely, and this did not change the results.

For correlation analyses Pearson’s correlation analysis was performed.

Statistical analyses were done with R statistical software version 2023.06.0 for Windows. A significance level of 0.05 was used for all hypothesis testing.

Plasma concentrations of C-peptide and glucose have been published previously [22]. In this paper slightly different statistical analyses were performed. The initial analyses were done with SAS statistical software without exclusion of the two measurements from the non-fasted subjects, therefore our results differ slightly.

## Results

### Study participants characteristics

The study participants constituted a homogeneous group of 24 healthy males with normal BMI (22.9 ± 1.6 kg/ m^2^, mean ± SD), HbA1c (33.7 ± 2.2 mmol/mol) and fasting blood glucose levels (4.8 ± 0.4 mmol/L) (Table 1). Plasma concentrations at fasted condition were for glucagon 3.5 ± 1.6 pmol/L, GLP-1 4.0 ± 1.5 pmol/L, GIP 4 ± 2 pmol/L, and C-peptide 429 ± 90 pmol/L (Table 1).

**Table 1:** Demographic characteristics and baseline fasting values for the 24 healthy male study participants

**Gender**	**Male (all)**
**Age (years)**	26 ± 5
**BMI (kg/m**^**2**^**)**	22.9 ± 1.6
**HbA1c (mmol/mol)**	33.7 ± 2.2
**HbA1c (%)**	5.2 ± 0.2
**Fasting glucagon level (pmol/L)**	3.5 ± 1.6
**Fasting GLP-1 level (pmol/L)**	4.0 ± 1.5
**Fasting GIP level (pmol/L)**	4 ± 2
**Fasting C-peptide level (pmol/L)**	429 ± 90
**Fasting glucose level (mmol/L)**	4.8 ± 0.4

Data are mean ± SD. *N* = 24

Plasma concentrations of HbA1c, C-peptide and glucose have been published previously [22]

*BMI* Body mass index, *HbA1c* Glycated hemoglobin, *GIP* glucose-dependent insulinotropic polypeptide, *GLP-1* Glucagon-like peptide-1

### Alpha-, L- and K-cell secretion during the study day and night

**Fig. 2:**
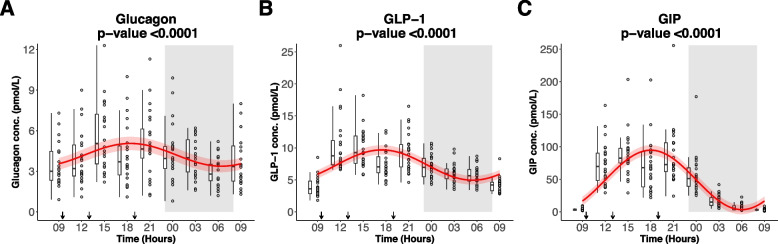
Diurnal rhythm of plasma concentrations of **A**) glucagon, **B** total GLP-1 and **C**) total GIP in 24 healthy males measured over 24 h. After an overnight fast the participants had 15 h of wakefulness (08 h-23 h) followed by a 9-h sleep opportunity (23 h-08 h) at the hospital ward. The sleep period is indicated with the grey area. The black arrows indicate time points for breakfast (9:30 h), lunch (13:00 h) and dinner (19:00 h). The red curves show the best fitting cosinor curve with confidence bounds for respectively **A**) plasma glucagon, **B** plasma total GLP-1 and **C** plasma total GIP. All three parameters fluctuate with a significant diurnal 24-h rhythm (all *p* < 0.0001). Plasma GIP fluctuates with the highest amplitude through day and night compared to plasma glucagon and plasma GLP-1. A boxplot and the full data set for respectively plasma glucagon, plasma GLP-1 and plasma GIP are also shown. The central box covers the 25^th^, 50^th^ and 75^th^ percentile. The whiskers marks 1.5*interquartile range (IQR). *N* = 22 for the first timepoint (*t* = 9 h), *N* = 24 for the rest of the remaining 8 time points. Conc.: concentration, GLP-1: Glucagon-like peptide-1, GIP: glucose-dependent insulinotropic polypeptide

Plasma concentrations of glucagon, GLP-1 and GIP fluctuated significantly with a diurnal 24-h rhythm, with the highest levels during the day and the lowest levels during the night (Fig. 2).

The 24-h diurnal rhythm of glucagon fluctuated around the rhythm adjusted average (mesor ± SD) of 4.2 ± 0.8 pmol/L with the relative amplitude of 19%. The cosinor analysis calculated the plasma concentration of glucagon peak clock time to be 18:26 h.

The 24-h diurnal rhythm of GLP-1 fluctuated around the rhythm adjusted average (mesor ± SD) of 7.3 ± 0.2 pmol/L with the relative amplitude of 33%. The peak clock time was calculated to be 17:28 h.

The 24-h diurnal rhythm of GIP fluctuated around the rhythm adjusted average (mesor ± SD) of 49 ± 2 pmol/L with the relative amplitude of 93%. The peak clock time was calculated to be 18:01 h.

Plasma levels of GIP fluctuated with the numerical largest rhythmic fluctuations compared to both plasma levels of glucagon and GLP-1.

Plasma glucagon, GLP-1 and GIP had estimated peak times *before* the participants were served dinner at 19:00 h.

### Beta-cell secretion and glucose levels during the study day and night

**Fig. 3:**
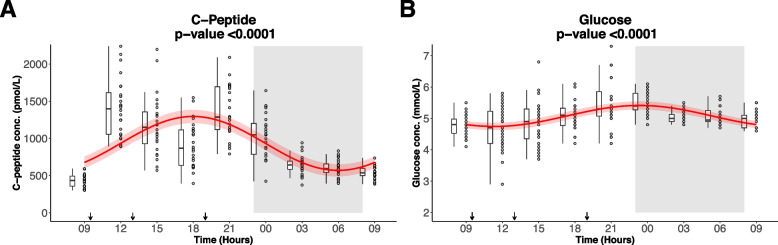
Diurnal rhythm of plasma concentrations of **A**) C-peptide and **B**) glucose in 24 healthy males measured over 24 h. After an overnight fast the participants had 15 h of wakefulness (08 h-23 h) followed by a 9-h sleep opportunity (23 h-08 h) at the hospital ward. The sleep period is indicated with the grey area. The black arrows indicate time points for breakfast (9:30 h), lunch (13:00 h) and dinner (19:00 h). The red curves show the best fitting cosinor curve with confidence bounds for respectively **A**) plasma C-peptide and **B**) plasma glucose. Both parameters fluctuate with a significant diurnal 24-h rhythm. Plasma C-peptide fluctuates with the highest amplitude through day and night compared to plasma glucose. A boxplot and the full data set for respectively C-peptide and glucose are shown. The central box covers the 25th, 50th and 75th percentile. The whiskers marks 1.5*interquartile range (IQR). *N* = 22 for the first timepoint (*t* = 9 h), *N* = 24 for the remaining 8 time points. Plasma concentrations of C-peptide and glucose were earlier published in [22]. In this analysis a slightly different statistical analysis was performed. Conc.: concentration

Plasma concentrations of C-peptide and glucose fluctuated significantly with a diurnal 24-h rhythm (Fig. 3), with the highest levels during the day and the lowest levels during the night. Data have been published previously (13).

The 24-h diurnal rhythm of C-peptide fluctuated around the rhythm adjusted average (mesor ± SD) of 933 ± 25 with the relative amplitude of 39%. The peak clock time was calculated to be 17:59 h (Table 2).

**Table 2:** Diurnal rhythm of glucagon, GLP-1, GIP, C-peptide and glucose in 24 healthy males

			**Output from Cosinor analyses**				
	**Unit**	**Baseline fasting value Mean (SD)**	**Mesor (SD)**	**Amplitude (SD)**	**Peak, clock time**	**Cosinor *p***	**Relative amplitude *%***
**Glucagon**	pmol/L	3.5 (1.6)	4.2 (0.8)	0.8 (1.2)	18:26	< 0.0001	19
**GLP-1**	pmol/L	4.0 (1.5)	7.3 (0.2)	2.4 (0.3)	17:28	< 0.0001	33
**GIP**	pmol/L	4 (2)	49 (2)	45 (3)	18:01	< 0.0001	93
**C-peptide**	pmol/L	429 (90)	933 (25)	362 (35)	17:59	< 0.0001	39
**Glucose**	mmol/L	4.8 (0.4)	5.1 (0.04)	0.3 (0.05)	23:26	< 0.0001	6

Data were analyzed with Cosinor analyses, and all five parameters follow a significant diurnal 24-h rhythm (*p* < 0.0001). *N* = 22 for the first timepoint (*t* = 9 h), *N* = 24 for the remaining 8 time points. Plasma concentrations of C-peptide and glucose were earlier published in [22]. In this analysis a slightly different statistical analysis was performed

Amplitude: half the difference between the highest and lowest value of the fitted cosinor curve, Relative amplitude: 100*(amplitude/mesor). Plasma concentrations of C-peptide and glucose have been published previously [22]

*GLP-1* Glucagon-like peptide-1, *GIP* glucose-dependent insulinotropic polypeptide, *Mesor* rhythm adjusted average about which oscillation occurs

The 24-h diurnal rhythm of glucose fluctuated around the rhythm adjusted average (mesor ± SD) of 5.1 ± 0.04 with the relative amplitude of 6%. The peak clock time was calculated to be 23:26 h.

Plasma concentrations of glucose fluctuated with a relatively smaller numerical amplitude during night and day compared to both C-peptide, glucagon, GLP-1 and GIP.

### Correlations

**Fig. 4:**
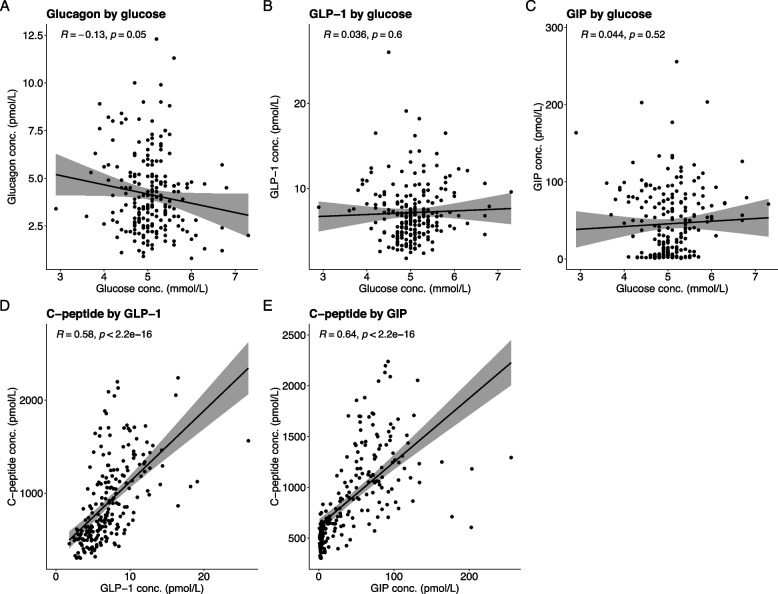
Pearson’s correlation analyses between plasma glucose and **A**) plasma glucagon, **B** plasma GLP-1 and **C**) plasma GIP and correlations between plasma C-peptide and **D**) plasma GLP-1 and **E**) plasma GIP in 24 healthy males measured over 24 h. There were no statistically significant correlations between plasma glucose and glucagon, GLP-1 and GIP, respectively. A numerical negative correlation was borderline significant for plasma glucose and plasma glucagon. There were significant correlations between plasma concentrations of C-peptide and GLP-1 and GIP. Conc.: concentration, GLP-1: Glucagon-like peptide-1, GIP: glucose-dependent insulinotropic polypeptide

To investigate if plasma concentrations of glucagon, GLP-1 and GIP depended on plasma concentrations of glucose rather than a circadian rhythm we performed correlation analyses. We did not observe statistically significant correlations between plasma glucose and glucagon, GLP-1, or GIP, respectively (Fig. 4). However, a numerical negative correlation was observed for plasma glucose and plasma glucagon (R^2^ = -0.13, *P* = *0.05)* (Fig. 4).

As GLP-1 and GIP contribute to glucose homeostasis by potentiating insulin secretion, we hypothesized that there would be a correlation between concentrations of GLP-1 and GIP and C-peptide. We found significant correlations between plasma concentrations of C-peptide and GLP-1 (R^2^ = 0.58, *p* < *0.0001*) and GIP (R^2^ = 0.64, *p* < *0.0001*) (Fig. 4).

## Discussion

Our results demonstrate that under meal conditions that are similar to that of many free-living individuals, plasma concentrations of glucagon, GLP-1 and GIP were observed to be higher during daytime and evening than night. As mentioned earlier, the human circadian system is inevitably affected by intake of food and vice versa [26] which is what makes the area interesting but also difficult to investigate [27].

Our results suggest that the increase in blood levels of both GLP-1 and GIP were higher after breakfast compared with after dinner even with almost identical distribution of macronutrients. Speculatively, this could be due to different physiologic responds to food during day and evening, however the results could be biased by the fact that the hormonal response might be different in a fasted state than in a well fed state and furthermore our blood sample frequency are not high enough to surely capture the peak or nadir values after a meal.

Normally, the peak levels of plasma glucagon, GLP-1 and GIP after a meal is around one hour [28, 29], this does however vary with the distribution of macronutrients in the meal and the size of the meal. Peak concentrations of glucagon, GLP-1 and GIP after meals were not evaluated in this study as blood samples were drawn with a 3-h interval. We know from previous studies that plasma levels of glucagon reaches baseline levels 3 h after a mixed meal [29] and GLP-1 and GIP still are slightly above baseline levels 4 h after a mixed meal [28, 29]. Like the peak levels, these time estimates do however vary with the distribution of macronutrients in the meal and the size of the meal. The measured plasma levels in our study are therefore inevitably affected by the meals. Even though, our data suggest that the response to a meal may be dependent on what time of the day the meal is eaten which could indicate a circadian affected rhythm of the secretion of these hormones.

Interestingly, the cosinor analyses calculated peak time of both plasma levels of glucagon, GLP-1, and GIP were around 18:00 h, before dinner was served at 19:00 h. Glucose peaks much later at 23:26 h which may be due to the lower levels of the measured hormones with blood glucose lowering effects late in the evening.

Like in our study, earlier studies have shown that the release of GLP-1 and GIP are more pronounced in the morning compared with the afternoon or evening [21]. Studies investigating night shift workers who eat during the night have also shown that the same meal eaten during the day and the night results in higher postprandial glucose concentrations during the night [30], which could be a result of lower circadian levels of glucagon and the incretin hormones during the night.

The lower levels of GLP-1 and GIP in the evening, could be an explanation for the often increased intake of food late in the evening. Other studies have shown that sleep deprivation increases appetite and compromise insulin sensitivity [31] and reduced sleep quality decreases GLP-1 blood levels and fullness score in the afternoon leading to increased food intake [32]. Furthermore, exposure of artificial light at late evening or at night which is part of our modern lifestyle affects the circadian rhythm [33] and may thereby affect the secretion of our metabolic hormones. This support findings of altered metabolic risk in patients with a disturbed circadian rhythm.

To our knowledge, a diurnal rhythm of glucagon in controlled settings using a validated ELISA has not previously been reported. Determinants of glucagon plasma levels are complex as several factors including glucose, GLP-1 and GIP regulates glucagon secretion. Whereas GLP-1 and glucose inhibit glucagon secretion, GIP stimulates its secretion [1]. Glucagon, GLP-1 and GIP fluctuated with the similar rhythmics although GIP had larger numerical differences compared to glucagon and GLP-1. Together, this may contribute to a circadian rhythm of glucagon in conjunction with the fact that a protein-rich meal may itself overrule any inhibitory actions of both glucose and GLP-1, since in a case of a protein-rich meal, amino acids stimulate the secretion of glucagon from the α-cell [2].

Because both incretin hormones lead to a glucose-dependent insulin secretion, we expected to find correlations between plasma concentrations of C-peptide and GLP-1 and GIP, which was also the case in our study. In contrast, we did not find correlations between glucose concentration and plasma levels of glucagon, GLP-1 or GIP, indicating that the diurnal changes in plasma levels of these three hormones are not merely reflecting meal intake but could also be regulated by a circadian rhythm either centrally or locally.

As earlier discussed, a major limitation to our study is the frequency of blood sampling, that may be insufficient to capture nadir and peak concentrations, for future studies it would be interesting to perform more frequent measurements. Another limitation is that the three meals during the day were not identical but relatively comparable, which might bias the response in measured hormones. A study with fixed amounts of macronutrients in all three meals during the day could rule out the bias of the different meals influencing the metabolic hormones in different ways. We do however believe that the meals in our study are similar to those of free-living individuals. For further studies it would be interesting to investigate whether the measured hormones in both obese patients and patients with T2D, follow the same fluctuations as reported here in healthy men. In addition, further studies should be done on women and elderly to increase the generalizability.

Furthermore, no sleep monitoring (e.g., with electroencephalogram (EEG)) or questionnaires were employed in this study to assess amount and quality of sleep during the allowed sleep time. Ideally the subjects would have come in for an EEG- or polysomnographic (PSG) monitored adaption night at the hospital ward the night before the study day. In this way, the fast could be controlled, the potential influence of the stress of meeting one hour earlier than the first blood sample could be excluded and the physiological reactions to food intake could be evaluated in relation to sleep quality and amount of sleep.

## Conclusions

Our results demonstrate that under meal conditions that are similar to that of many free-living individuals, plasma concentrations of glucagon, GLP-1 and GIP were observed to be higher during daytime and evening than overnight in young healthy men. This may have clinical implications related to timing of meals, disturbed circadian rhythm and the altered metabolic risk in patients with a disturbed circadian rhythm.

## Data Availability

Coding and raw-data are available through https://github.com/nicwin98/Circadian.

## Abbreviations

GLP-1: Glucagon-like peptide-1
GIP: Glucose-dependent insulinotropic polypeptide
TD2: Type 2 diabetes
ELISA: Enzyme-linked immunosorbent assays
BMI: Body mass index
HbA1c: Glycated hemoglobin
E%: Energy percent
K_3_EDTA: Ethylene diamine tetraacetic acid
HPLC: High performance liquid chromatography
Min: Minimum value
Max: Maximum value
IQR: Interquartile range
Conc.: Concentration
EEG: Electroencephalogram
PSG: Polysomnography
